# Miura-ori structured flexible microneedle array electrode for biosignal recording

**DOI:** 10.1038/s41378-021-00259-w

**Published:** 2021-07-21

**Authors:** Yue Hou, Zhaoyu Li, Ziyu Wang, Hongyu Yu

**Affiliations:** 1 Department of Mechanical and Aerospace Engineering, The Hong Kong University of Science and Technology, Kowloon, Hong Kong, SAR 999077 China; 2 The Institute of Technological Sciences, Wuhan University, Wuhan, 430072 China

**Keywords:** Electrical and electronic engineering, Nanoscience and technology

## Abstract

Highly reliable signal recording with low electrode-skin impedance makes the microneedle array electrode (MAE) a promising candidate for biosignal sensing. However, when used in long-term health monitoring for some incidental diseases, flexible microneedles with perfectly skin-tight fit substrates lead to sweat accumulation inside, which will not only affect the signal output but also trigger some skin allergic reactions. In this paper, a flexible MAE on a Miura-ori structured substrate is proposed and fabricated with two-directional in-plane bendability. The results from the comparison tests show enhanced performance in terms of (1) the device reliability by resisting peeling off of the metal layer from the substrate during the operation and (2) air ventilation, achieved from the air-circulating channels, to remove sweat. Bio-signal recordings of electrocardiography (ECG), as well as electromyography (EMG) of the biceps brachii, in both static and dynamic states, are successfully demonstrated with superior accuracy and long-term stability, demonstrating the great potential in health monitoring applications.

## Introduction

**Fig. 1:**
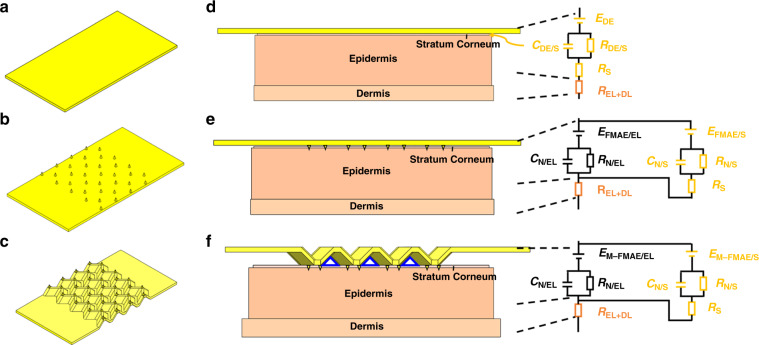
Schematic drawing and working principle of the flexible DE, flexible MAE, and M-MAE. Schematic of the (**a**) flexible dry electrode, (**b**) flexible MAE, and (**c**) Miura-ori structured MAE, together with their equivalent circuits in (**d**), (**e**) and (**f**), respectively

Stable signal extraction in the long term is an essential part of what makes an electrode suitable for biosignal recording. For example, in the early diagnosis of heart disease, ECG is the standard diagnostic method for monitoring ventricular contraction and relaxation. During this test, conventional wet Ag/AgCl electrodes are placed on the patients’ chest. However, for most cases, the abnormal pulse does not appear on the record sheet during this 1-min test, and it is necessary to have long-term monitoring in home care. In this case, wet Ag/AgCl electrodes are no longer suitable since the electrolytic gel on top of the electrodes will not only increase the electrode-skin interface impedance (EII) as it gradually becomes dry but also block sweat evaporation and affect signal acquisition^1^. Therefore, some substitutes have been proposed by researchers, such as dry electrodes (DEs) or flexible DEs^2,3^, silicon-based microneedle array electrodes (MAEs)^4^, and flexible MAE (FMAEs)^5–7^. These electrodes, such as flexible DEs (Fig. 1a), can be easily prepared, but poor attachment to the skin sometimes induces an air dielectric layer between the electrode and the skin, causing a huge contact resistance. For the MAEs and FMAEs (Fig. 1b), although they can solve most of the issues mentioned above, their fabrication sometimes relies on the MEMS-based process of photolithography and Si etching^8^, which presents high costs.

Human skin consists of three layers, which, from the outside to the inside, are the stratum corneum layer (SCL), epidermis layer (EL), and dermis layer (DL)^9^. The SCL consists of dead cells and is surrounded by sweat and grease. Due to the lack of transport channels for ions, the SCL has high electrical impedance^10^. As shown in the equivalent circuit in Fig. 1d, when the skin is directly in contact with a DE, the DE and sweat or grease inside the SCL form a metal-electrolyte interface and develop a half-cell potential *E*_DE_, while *C*_DE/S_ and *R*_DE/S_ are caused by the polarization effect and *Rs* represents the resistance of sweat. Below the SCL, there are the EL and DL, which share similar electrical properties, where ions are carried by an aqueous medium, which is considered as *R*_EL+DL_. By piercing through the SCL and reaching the EL, as shown in Fig. 1e for MAEs and FMAEs, the SC layer’s high electrical impedance can be eliminated^11^. In this circumstance, the microneedles and SCL form a metal–electrolyte interface with *E*_FMAE/EL_, *C*_N/EL_ and *R*_N/EL_. Moreover, these microneedles maintain a stable connection with the skin after penetration and help produce reliable biosignals with high resolution^12^. However, MAEs or FMAEs also have drawbacks; for instance, the tight attachment between the flat substrate area and skin still leads to the accumulation of sweat and grease, which will jam the sweat glands and affect the signal output around the microneedle tips. In addition, a long-term skin-tight attachment may cause skin allergic reactions^7,13,14^.

To address the above problem, we fabricated a Miura-ori structured microneedle array electrode (M-MAE) with induced ventilation channels inside (Fig. 1c). For the M-MAE, a mold casting fabrication method with computer numerical control multiaxis machined molds is employed. This Miura-ori structure has been widely used in flexible and stretchable electronic devices due to its unique deformability and attachment-enhancing capability during stretching and bending^15,16^. When the M-MAE patch is applied to the skin for ECG or EMG testing, the microneedles on top pierce the skin for signal acquisition, and the Miura-ori structure ensures the bonding integrity between the top metal layer and the substrate under bending, which is also of vital importance in the biosignal sensing process. Additionally, the electrode systems’ Miura-ori tessellation pattern provides air-circulable channels (indicated by blue triangles) in the structure to help ventilate sweat away (Fig. 1f).

In this work, we describe a novel low-cost and high-volume production compatible fabrication method combining high-precision machining and PDMS mold pressing together to directly form the microlevel needle tips with the macrolevel Miura-ori substrates. The outstanding performance of our flexible M-MAE was comprehensively analyzed compared to the conventional flexible MAE, flexible DE, and commercial wet Ag/AgCl electrode in a series of carefully designed tests. The M-MAE can be bent in the in-plane direction in two directions with a large bending degree (bending radius of 1 cm) and a small self-resistance variation. At the same time, the main feature of the Miura-ori structured air ventilation channels endows the needle patches with stable signal output by removing sweat in time. Moreover, in situ recording of ECG biosignals (both in static and dynamic states), as well as the EMG signal produced on the biceps brachii, was performed with the proposed M-MAE and analyzed.

## Results and discussion

### Fabrication process

**Fig. 2:**
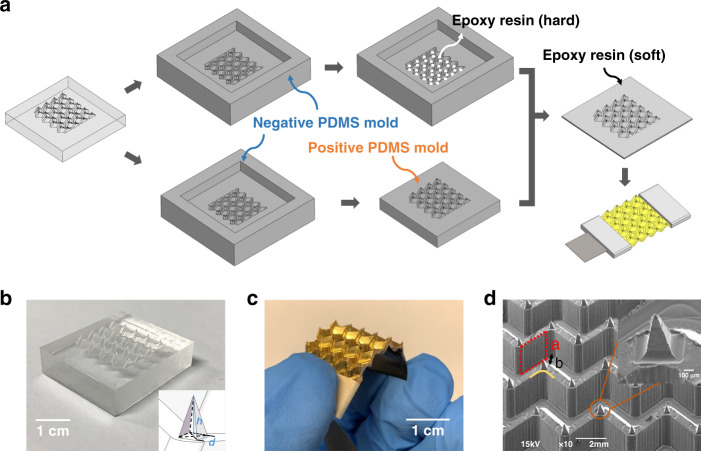
Fabrication process of the M-MAE. **a** Schematic of the fabrication process of the Miura-ori structured microneedle array electrodes. **b** Optical image of the acrylic mold (inset is the predesigned structure for a single microelectrode). **c** Optical image of the flexible Miura-ori structured microneedle electrode sample. **d** SEM image of the Miura-ori structured electrode sample (inset is the enlarged SEM image of a single microneedle)

A computer numerical control multiaxis machine was employed to fabricate acrylic block molds for Miura-ori structured microneedle array electrodes with precisely parallel milling contours. The detailed machining process of all the acrylic microneedle molds is provided in the supporting information Fig. S1. We chose acrylic material in this experiment since it can be easily separated from PDMS after curing without additional surface treatment. The fabrication methods of the positive and negative PDMS molds were reported in previous research^17^. As shown in Fig. 2a, two types of epoxy resins were used to fill the cavity of the negative mold successively; the first was a hard epoxy resin to fill and form the microneedle tips, followed by a soft resin on top to form the flexible Miura-ori structured substrate by the mold pressing method. After curing in air for 12 and 24 h, the sample was cut on both sides to expose the ventilation channels. Finally, to functionalize its electrode conductivity, the sample was coated with 50 nm titanium and 200 nm gold metal layers to ensure its conductivity. At the design stage of the acrylic mold, a 3D model was first drawn using SOLIDWORKS software. The microneedle was designed as a rectangular pyramid (shown in the inset of Fig. 2b), and the bottom-based width *d* and height *h* of this rectangular pyramid were designed to be 350 μm and 700 μm. The finished M-MAE sample is demonstrated in Fig. 2c with a size of 28 mm (± 0.5 mm) × 16 mm (± 0.5 mm) × 1 mm (± 0.25 mm) (length × width × sample thickness). The M-MAE morphology is shown in Fig. 2d, and the zoomed-in picture shows a single microneedle. The average height, tip radius, and base width for this M-MAE are approximately 630 ± 46 μm, 16 ± 2 μm and 380 ± 32 μm, respectively. Compared with other research works, this design geometry is similar and suitable for skin penetration^18,19^. The bottom Miura-ori structure, as shown in Fig. 2d, consists of many rhombi with a side length of 1.80 mm (indicated in red dotted frame) and a patterned distance *b* of 0.65 mm. Unlike the traditional Miura-ori structure, this patterned distance is enlarged to allow the microneedles to sit on top and increase the volume for the bottom ventilation channels. All the connecting regions (yellow line in Fig. 2d) were designed with rounded corners to help release localized stress concentrations of the top metal layers, and a detailed drawing of M-MAE is provided in the supplementary information (Fig. S2).

### EII testing

**Fig. 3:**
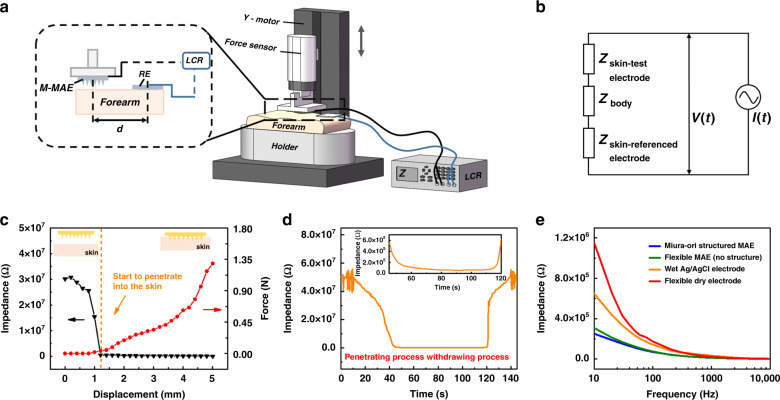
EII tests for M-MAE, FMAE, WE, and FDE samples. **a** Schematic drawing of the EII test setup. **b** Equivalent circuit for the EII testing. **c** EII and compression force measurement of the M-MAE. **d** EII testing for a whole cycle of the M-MAE insertion and withdrawal process. **e** EII under input current frequencies from 10 Hz to 10,000 Hz for M-MAE, FMAE, WE, and FDE samples

The electrode-skin interface impedance was tested using the two-electrode method with the test setup shown in Fig. 3a^20^. As shown in Fig. 3b, the equivalent circuit of this EII test included two-electrode-skin contact impedances *Z*_skin-test electrode_, and *Z*_skin-referenced electrode_, and the inner body impedance *Z*_body_. The M-MAE was first fixed on the customized plate, which was clamped by the upper force sensor, and a wet Ag/AgCl electrode was used as the reference electrode (RE) which was directly placed on the forearm. The distance between these two electrodes was strictly set as 4 cm, and the input current frequency was 1 kHz. The M-MAE was first stopped at a distance of 1.2 mm from the skin surface and then gradually moved down to pierce the forearm. This process was driven by the *y*-axis sliding shaft controlled by a handwheel, and the penetration force and EII were recorded simultaneously, as shown in Fig. 3c, at a sampling distance of 0.2 mm. A whole test cycle in the time domain, which included penetration and withdrawal of the M-MAE, is recorded in Fig. 3d. From the experimental results, the EII decreased by two orders of magnitude, from the 10^7^ Ω level to the 10^5^ Ω level, when the M-MAE touched the SCL, and this value then gradually dropped to the 10^4^ Ω level (shown in the zoomed-in data in Fig. 3d) as the M-MAE gradually pieced through the EL and reached the DL. Skin images of the residual pinhole traces and test details are provided in the supplementary information (Fig. S3). The EII under the input current frequency from 10 Hz to 10 kHz was measured under a constant force of 0.3 N (Fig. 3e). Here, samples of the flexible MAE and flexible DE with the same detection surface area (not considering the area for the connection of conductive cloth tape) of 16 mm (± 0.5 mm) × 16 mm (± 0.5 mm) were fabricated and together with commercial wet Ag/AgCl electrodes, were used for comparison. For these four types, the EII decreased with increasing input current frequency. Compared with the EII test results for the wet Ag/AgCl electrode and flexible dry electrode, samples with microneedles on top showed outstanding smaller impedance, almost half that of the wet Ag/AgCl electrode and one-third that of the dry electrode, which corresponds well to the working principle of the MAE mentioned in the previous section. The results for the MAEs with and without the Miura-ori substrate were also compared. The slightly smaller EII with the change in the input frequency illustrated that the M-MAE sample was competitive with the traditional flexible MAE.

### Flexibility testing

**Fig. 4:**
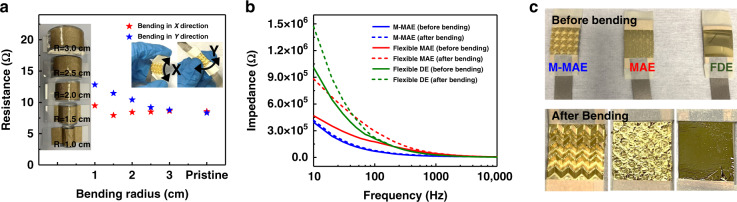
Flexibility tests for M-MAE. **a** Self-resistance of the M-MAE under different bending radii in *x* and *y* bending directions. **b** EII measured for the M-MAE, flexible MAE, and flexible DE before and after bending. **c** Surface images of the M-MAE, flexible MAE, and flexible DE before and after bending

To investigate the flexibility of the M-MAE, we prepared five laser-cut acrylic half-cylinders with different bending radii (*R*) from 1 cm to 3 cm, with 0.5 cm intervals in between, to test the sample resistance in bending situations. As shown in the inset of Fig. 4a, a piece of double-sided adhesive conductive cloth was stuck on the top of the half-cylinder. During each test, the needle tips were directly attached to the center of the conductive cloth. Compared with a more simplified out-of-plane 3D wavy structure, the Miura-ori structure enables two-directional bending without any damage (given in the *x*- and *y*-directions in the inset of Fig. 4a). Two M-MAE sample resistances were recorded under different bending radii in the *x*- and *y*-directions, and the experimental details are provided in Fig. S4. Fig. 4a demonstrates good stability for bending in the x-direction, with the self-resistance varying from the original state of 8.56–9.48 Ω at the largest bending degree (bending radius of 1.0 cm), while in the *y*-direction, the self-resistance increases from the original state of 8.33–12.82 Ω (bending radius of 1.0 cm). The self-resistance is negligible under bending compared with the EII, thereby ensuring the accuracy of the measurement. For biosignal recording, the bending radius is usually small in one direction (i.e., y-direction) and large in the perpendicular direction (i.e., *x*-direction), such as when performed on a finger, arm, or elbow, so the M-MAE can be adjusted to suit the skin curvature, thereby realizing suitable attachment with stable self-resistance.

To compare the stability under bending situations, the EII as a function of the input current frequency of the three bendable electrodes, namely, the M-MAE, FMAE, and flexible DE, were tested before and after ten bending cycles on the 3D printed mold with a bending radius of 2 cm, and the test method was the same as in the previous section. As shown in Fig. 4b, the EII measured for the M-MAE was slightly increased by 5.15% after bending, while for the flexible MAE and DE, larger increases of 87.98% and 45.60%, respectively, were obtained. The surface conditions of all three samples before and after bending are shown in Fig. 4c, which can also account for the variation difference in Fig. 4b. Both the microlevel parallel milling contours and the Miura-ori structure contribute to the excellent adhesion between metal layers and the epoxy resin. For the other two patterns, metal layers were detached from their epoxy substrates after bending, embodied as newly formed surface metal buckling, which directly led to the dramatic increase in the skin-electrode impedance.

### Ventilation testing

**Fig. 5:**
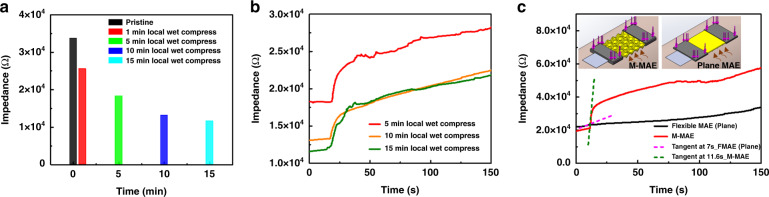
Ventilation testing for M-MAE and flexible plane MAE. **a** EII measured under different local wet compression treatment times of 0, 1, 5, 10, and 15 min. **b** Ventilation testing of the M-MAE after local wet-compression treatment for 5, 10, and 15 min. **c** Ventilation testing for M-MAE and flexible plane MAE after 5 min of wet-compression treatment

The EII under different wet conditions was tested on the forearm to evaluate the effect of sweat on the M-MAE. First, four pieces of cleanroom paper of 2 cm × 2 cm were dipped into 0.5 ml artificial sweat for 1 min before being successively applied to the test region of skin, which was 4 cm from the center of the referenced wet electrode, for 1, 5, 10, and 15 mins, successively. After that, the M-MAE was fixed on the test region under a compression force of 0.3 N, and a hot air blower was also fixed at a distance of 10 cm with a height adjusted to be relatively the same as that of the ventilation channels of the M-MAE. The original EII at 1 kHz before turning on the blower is collected in Fig. 5a, with the wet-compression time increased, which represented sweat accumulation, the EII gradually dropped from the pristine state value of 3.37 × 10^4^–1.16 × 10^4^ Ω (application for 15 mins). The EII variation of the M-MAE under different wetness conditions after turning on the blower is presented in Fig. 5b, which included two distinctive stages. After turning on the blower, a sudden increase could be found in the first 5 s. With the ventilation channels embedded in the M-MAE, the hot air rapidly took the extra water molecules away during this period. After that, the skin moisture began to evaporate under the hot and dry wind, leading to the subsequent growth of the resistance at a low rate. Cloth tape was used to cover the skin area except for the test region to reduce the interference. Ventilation tests were also conducted on the conventional MAE under the same wet-compression treatment of 5 mins. As shown in Fig. 5c, the effectiveness of the ventilation channels is quite remarkable when comparing the tangent lines at their first growth point; the striking increase of the EII resulted in a rising slope of 8259.25 Ω/s for the M-MAE, while for the flexible MAE, the impedance increased slowly with a slope of 289.50 Ω/s due to the gradual evaporation process from the skin under the hot surrounding environment.

Ventilation testing was conducted to visualize the effect of accumulated sweat on electrodes. Water molecules and conductive ions such as K^+^ and Cl^-^ in sweat will increase the conductive path that connects the electrode with the EL and DL and reduce the EII, causing instability in biosignal sensing. However, with the ventilation channels, air convection occurs occasionally, which can postpone or even prevent sweat accumulation in the long run.

### ECG and EMG signal recording

**Fig. 6:**
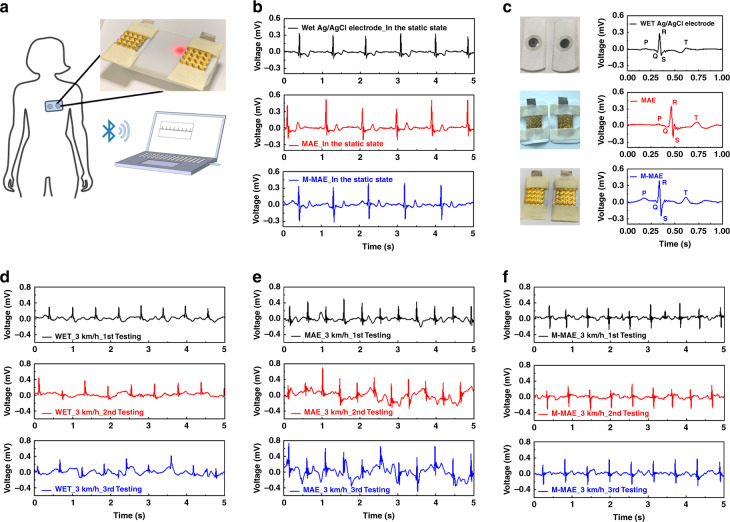
The ECG signal recording for both the static and dynamic states with the wet Ag/AgCl electrodes, MAEs, and M-MAEs. **a** Setup for the static ECG signal recording. **b** Static state ECG signals recorded by the wet Ag/AgCl electrodes, MAEs, and M-MAEs. **c** Assembled wet Ag/AgCl electrode, MAE, and M-MAE images with zoomed-in ECG signal for a single characteristic signal. Dynamic state ECG signals recorded in the presence of sweat at a constant walking speed of 3.0 km/h three times at an interval of 10 mins after running at a speed of 8.0 km/h for (**d**) wet Ag/AgCl electrodes, (**e**) MAEs, and **f** M-MAEs

A pair of M-MAEs was applied for ECG signal testing to verify the M-MAE’s practicability and performance in real-time biosignal recording. First, the conductive Ag/AgCl gel was removed from the traditional ECG button patches and substituted by our M-MAEs. As demonstrated in Fig. 6a, the portable ECG sensor with M-MAEs was fixed in front of the heart by elastic tape wrapped around the chest. Here, the ECG signal was first recorded and further processed by a BMD101 chip, which has an accurate ECG raw data output. Then, it was transferred to the computer by a Bluetooth module (RTL8762AG). The static ECG signals in the time domain were recorded using the M-MAEs when the volunteer was lying down. Two pairs of commercial wet Ag/AgCl button patches and MAEs were also tested at the same test spots for comparison. As shown in Fig. 6b, compared with the wet patches, the signals acquired from electrodes with microneedles on top demonstrated a larger amplitude with a more distinct ECG characteristic wave due to their stable penetration into the outermost SC layer (Fig. 6b). For better visibility, zoomed-in figures for a single characteristic wave from all electrodes are provided in Fig. 6c. As presented in this figure, when using all three electrodes, the ECG typical features, namely, the P wave, QRS complex, and T wave, could all be recognized from their distinguishable waves.

Furthermore, ECG signals were also tested in a dynamic state. The same volunteer was asked to run at a speed of 8.0 km/h for 10 mins, followed by constant walking at 3.0 km/h for another 5 mins, and the data were collected during the walking interval for all three types of electrodes. The long-running time at high speed ensured that sweat was produced, and the sweat could be seen on the volunteer’s outer skin after the first run. The test results with different electrode pairs are provided in Fig. 6d–f. The test results of M-MAE (Fig. 6f) exhibit better dynamic performance, as indicated by the following three points: (1) a larger peak-to-peak amplitude at all speeds (expected value of |*V*_max_-*V*_min_|), compared with Ag/AgCl electrodes, (2) smaller signal drift in the sweaty situation (square deviation for ${{\left| {V_{{\rm{max}}} + V_{{\rm{min}}}} \right|}}/{2}$) when compared with both the wet electrodes and MAE, and (3) much more complete P, and T waves and QRS complex when compared with the wet Ag/AgCl electrodes and MAEs. All statistical analyses for the ECG test for both the static and dynamic states are provided in the supplementary information as Fig. S5 and Fig. S6.

**Fig. 7:**
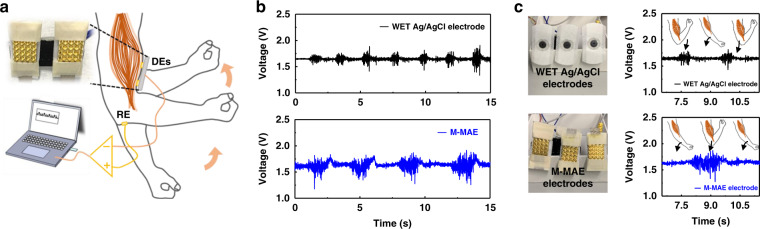
The EMG signal recording with the wet Ag/AgCl electrodes and M-MAEs. **a** Setup for the static EMG signal recording. **b** EMG signals recorded by the wet Ag/AgCl electrodes and M-MAEs. **c** Assembled electrode images, of wet Ag/AgCl electrodes and M-MAEs with zoomed-in EMG signals for two arm-bending cycles

As shown in Fig. 7a, the EMG signal from the biceps brachii was also tested. Three electrodes, which included a pair of detection electrodes (DEs) and one reference electrode (RE), were placed on the arm to test the biosignal from the biceps brachii. During testing, wet Ag/AgCl electrodes and M-MAEs were used separately for this experiment. As shown in Fig. 7b, both M-MAEs and traditional wet patches were used to obtain EMG signals of the biceps brachii. Raw data were collected during ten cycles of an arm lifting up (120 degrees to exert muscle power) and laying down (relaxing state) process. During each cycle, the muscle power exertion corresponded to the sudden increase of the signal intensity shown in Fig. 7c on the right, and the result obtained with M-MAEs demonstrates a better signal output with a larger amplitude as well as a larger signal-to-noise ratio (detailed analysis is provided in Fig. S7).

Regarding the ECG and EMG signal recording results obtained with the wet Ag/AgCl and M-MAEs above, those obtained with the M-MAEs demonstrated a better biosensing quality than those obtained with the commercial Ag/AgCl wet patches, especially for sweaty dynamic measurement, which, along with its unique reusability and long-term stability, proves the superiority of the M-MAE in the field of real-time biosensing.

## Conclusion

In summary, a novel flexible MAE with a Miura-ori pattern was fabricated for accurate and stable biosignal monitoring. High-precision machining was used to directly form the mold, which includes both the microlevel needle tips and a bottom macrolevel Miura-ori structure. A PDMS mold pressing process was also applied to extrude the flexible M-MAE patches. The comparison test of the EII and flexibility between different electrodes demonstrate the superiority of this minimally invasive M-MAE in biosignal sensing due to its much smaller skin-electrode impedance and a much more stable sensing ability for bending. Furthermore, ventilation testing of the M-MAE validates its airiness improvement with air-permeable channels, which helps remove sweat in time to maintain stable signal output. To verify the in situ practicability and long-term stability of our structural microneedle electrode, real-time biosignal recording of ECG and EMG with M-MAE sensing patches was also tested. All the test results show the advantages of the M-MAE in the field of long-term biosignal sensing.

## Materials and methods

### Sample preparation

A negative mold was demolded from an acrylic block using PDMS (DC SYLGARD 184, base: curing agent =1:1, cured after 6 h on a hot plate at 60 °C). The surface of the top mold was exposed to trichloro(1*H*,1*H*,2*H*,2*H-*perfluorooctyl) silane (Sigma-Aldrich) (100 μL) in a vacuum chamber for 6 h to help demold the negative PDMS mold from the positive mold. Hard epoxy resin (SHENZHEN JINHUA ELECTRONIC MATERIAL CO., LTD, Model No. 1201AB-3, base: curing agent = 1:1) was used to fill the needle tips, while soft epoxy resin (SHENZHEN JINHUA ELECTRONIC MATERIAL CO., LTD, Model No. 607AB-5, base: curing agent = 3:1) was used as the Miura-ori substrate. These two epoxy resins were officially tested and proved to be nontoxic. Before curing at room temperature for 6 h and 12 h, respectively, these two epoxy resins were placed in a vacuum chamber to release the air bubbles for 15 min. Ti (50 nm) and Au (200 nm) were coated on the flexible M-MAE in sequence by an ARC-12M sputtering system from the Nanosystem Fabrication Facility (NFF) at the Hong Kong University of Science and Technology. For the ventilation testing, artificial sweat (SZZW-HY-4.7) was bought from SHENZHEN ZHONGWEI EQUIPMENT Co., LTD to imitate sweat accumulation.

### Characterization

SEM was used to characterize the M-MAE sample surface morphology and test the parameters of the microneedle tips. The electrode-skin impedance was tested with a VICTOR LCR 4091A Digital Bridge, and the compression force during penetration was evaluated from the setup, which contained a precise tension gauge with a calibrated scale system (HANDPI). ECG signals were recorded through an Arduino UNO, and the raw data of the EMG signal was recorded from a data acquisition card from National Instruments (NI).

All skin tests, including the wearing Microneedle electrodes for signal acquisition and sweat tests, were conducted with all the participants’ consent.

## Supplementary information

Supplemental Material

Dataset for all figures
