# Supplementary material for: Miura-ori structured flexible microneedle array electrode for biosignal recording
Source: Microsyst Nanoeng. 2021 Jul 21;7:53. doi: 10.1038/s41378-021-00259-w (PMC8433437; doi:10.1038/s41378-021-00259-w)
Supplement: Supplementary file 1 — Supplemental Material [file 41378_2021_259_MOESM1_ESM.docx]

# Supplementary Information

# Miura-ori structured flexible microneedle array electrode for biosignal recording

*Yue Hou^1^, Zhaoyu Li^1^, Ziyu Wang^2^*, Hongyu Yu^1^**

1 Department of Mechanical and Aerospace Engineering, The Hong Kong University of Science and Technology, Kowloon, Hong Kong SAR 999077, China

2 The Institute of Technological Sciences, Wuhan University, Wuhan 430072, China

**Corresponding authors:**

Prof. Hongyu Yu

Email: [hongyuyu@ust.hk](mailto:hongyuyu@ust.hk)

Telephone: +852 34692754

Prof. Ziyu Wang

The Institute of Technological Sciences, Wuhan University, Wuhan 430072, China

Email: [zywang@whu.edu.cn](mailto:zywang@whu.edu.cn)

Telephone: +86 13886191929

**Fabrication Process of the Acrylic Mold**


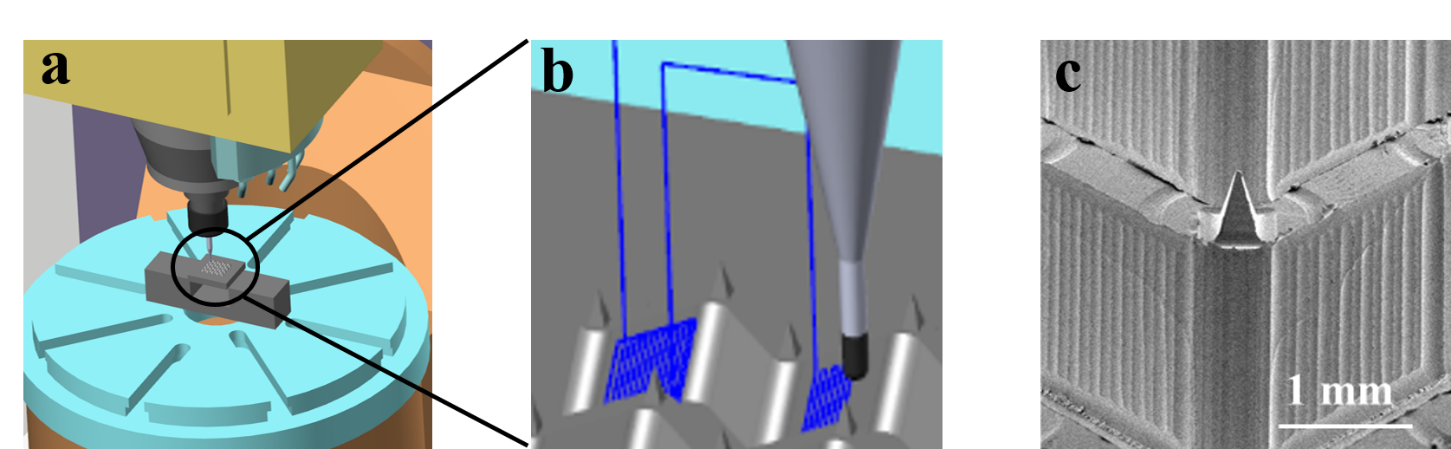


**Fig. S1 a** Schematic of the five-axis machining process of the M-MAE acrylic mold. **b** Milling contour for the Miura-ori structure. **c** Surface structured form from the milling machine.

The Numerical Controlled (NC) milling machining method was used for machining the whole structure of the acrylic mold, and the physical cutting process was conducted on a JINGDIAO JDGR200 five-axis CNC machine tool (**Fig. S1 a**). The machine tool's motion axes can move simultaneously so that the cutter can conduct the unique spatial movement along a predefined path (**Fig. S1 b**) for removing the material from the raw workpiece, leaving the finished designed model. The milling cutter moves periodically from one defined point to another, leaving the parallel cutting trail in every parallelogram of the Miura-ori structure (**Fig. S3 b**). The uneven surface also improves the attachment of the metal layers and the bottom substrate.

**Detailed Drawing of the M-MAE**

**
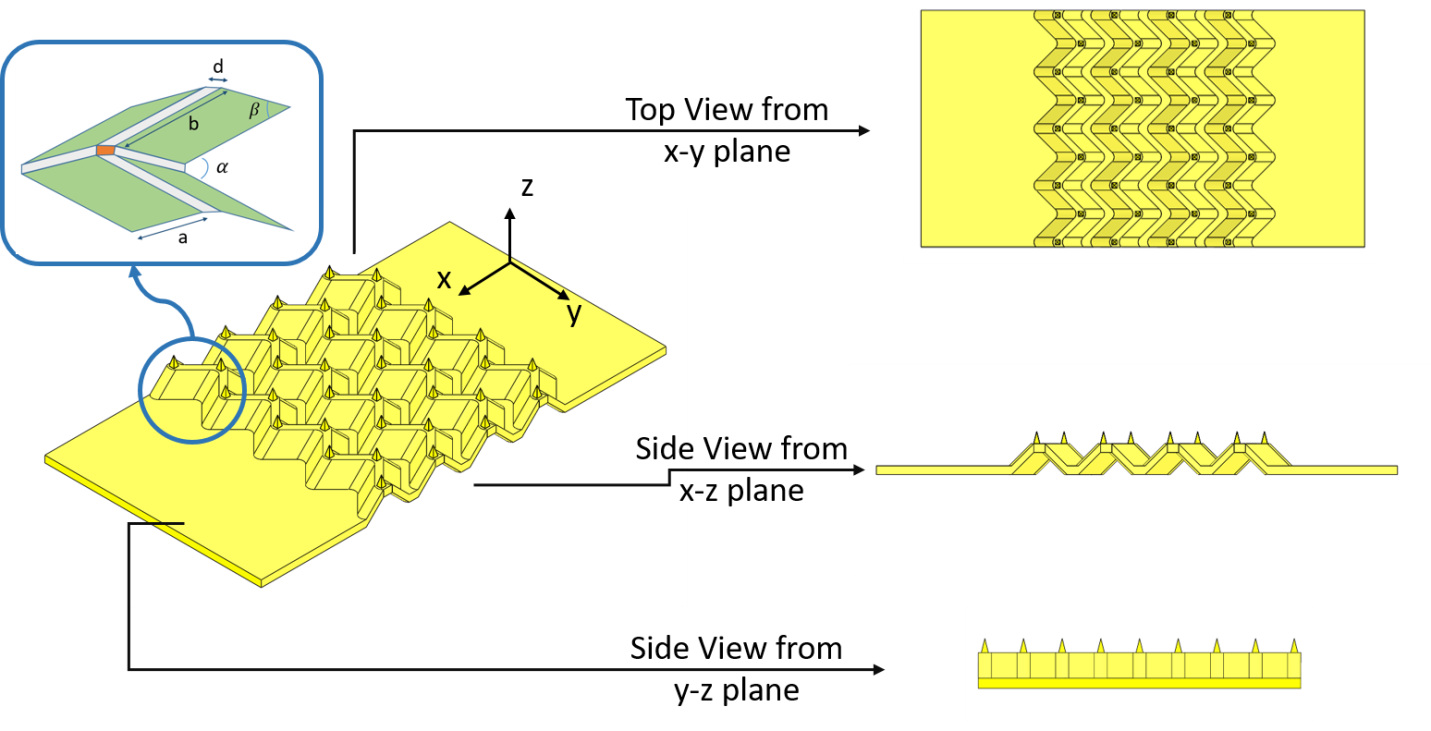
**

**Fig. S2** Schematic illustration for the Miura-ori structure microneedle array.

The Miura-ori structure is used as the substrate for the microneedle electrode array. For the traditional Miura-ori structure (demonstrated as the green color in the inserted figure in **Fig.S2**), its geometry can be defined only when the two side lengths a, b and two angles $\alpha, \beta$ are given^1^. Here, a, b, $\alpha, \beta$are set as 1.8 mm, 1.8 mm, 90 degrees and 60 degrees, respectively. In order to place the microneedle array on top, a quadrate platform (actually with a rounded corner) is designed with a side length d of 0.65 mm. Besides, the detailed drawings with both top view and two side view figures of the M-MAE are provided in **Fig. S2**.

**Skin Images of M-MAE Testing**


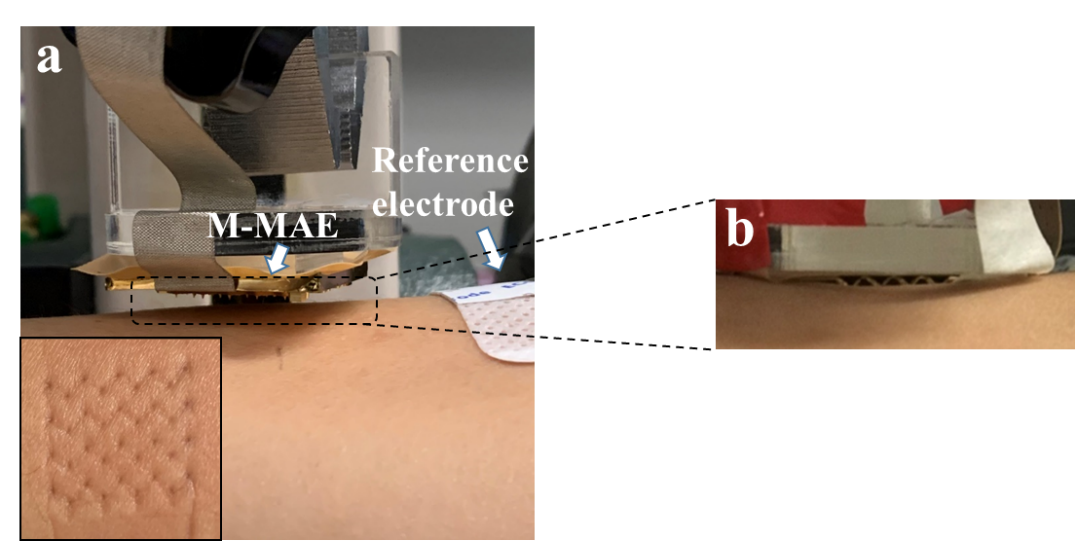


**Fig. S3** **a** Schematic of M-MAE skin testing (Inserted in is the pinhole image after withdrawing the microneedles). **b** Cross-section image of the M-MAE penetrated the skin.

When the M-MAE pierce the skin, the volunteer could feel very slight pain, which also proves the microneedle's penetration into the DL layer where the nerve system exists. And the left pinholes after withdrawing the microneedle were provided in **Fig. S3 a**. No bleeding or swelling situation had been found during the whole testing, and the traces of the pinhole would disappear around ten to fifteen minutes. The M-MAE cross-section view that penetrated the skin had clearly demonstrated the ventilation channels from the Miura-ori structure (**Fig. S3 b**).

**Experiment Detail of the Bending Test**


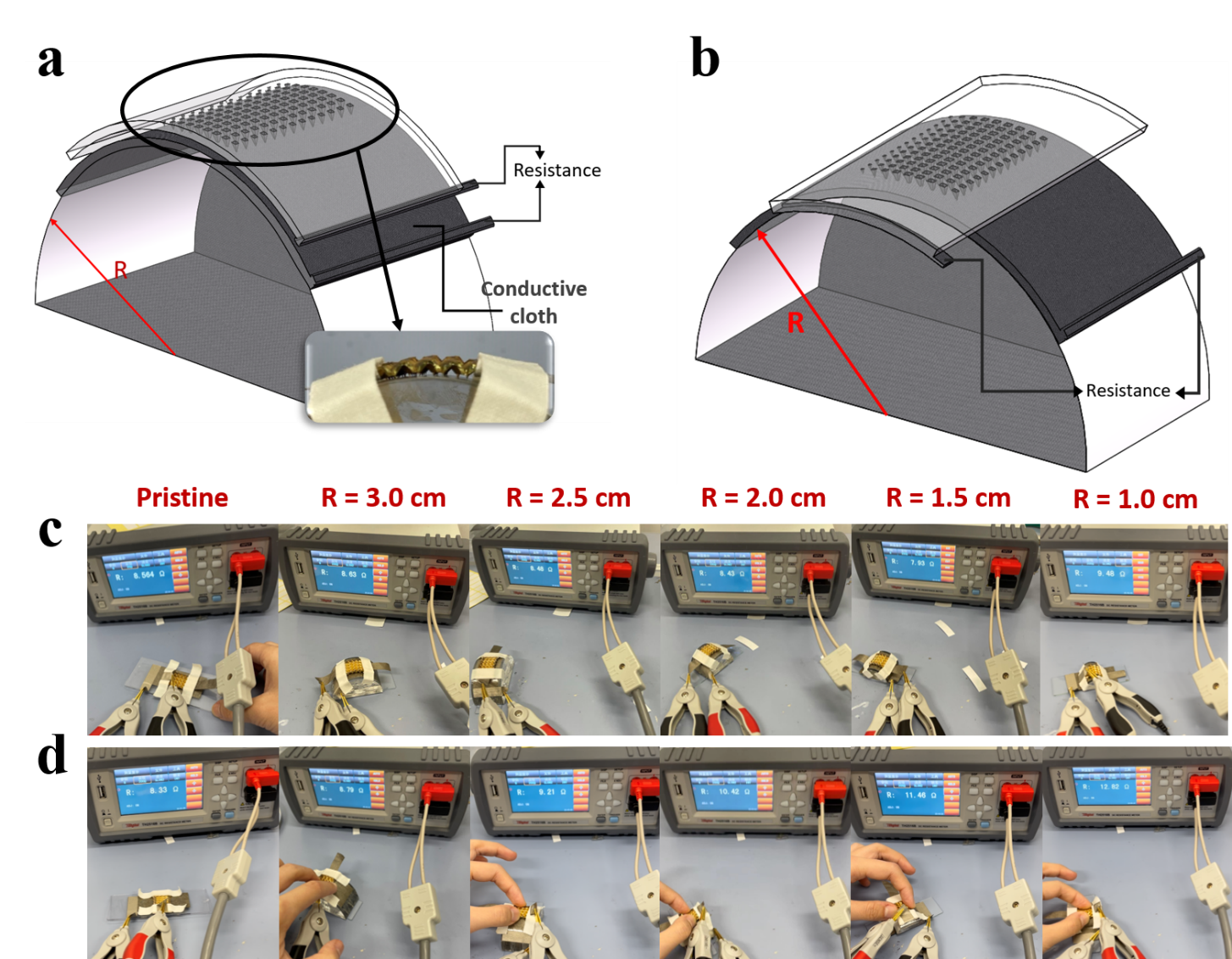


**Fig. S4** The schematic diagram of the bending testing of M-MAE in **a)** x-direction and **b)** y-direction in different bending radii (R) from 1 cm to 3 cm, with 0.5 cm intervals in between.

As presented in **Fig. S4**, the bending tests in x-direction and y-direction were conducted manually by folding the sample in different directions to fit the laser-cut acrylic half-cylinders. When testing in the x-direction (**fig. S4 a**), we used cloth tape to fix the sample on two sides to ensure that the needle tips firmly touched the conductive adhesive cloth tape before testing the resistance. In the testing of y-direction (**fig. S4 b**), the sample was bent manually to fit the changing curved surface. Based on this method, the resistance between the microneedle tips and one of the lead during the bending process could be tested.

**Data Analysis for ECG and EMG Testing**

**
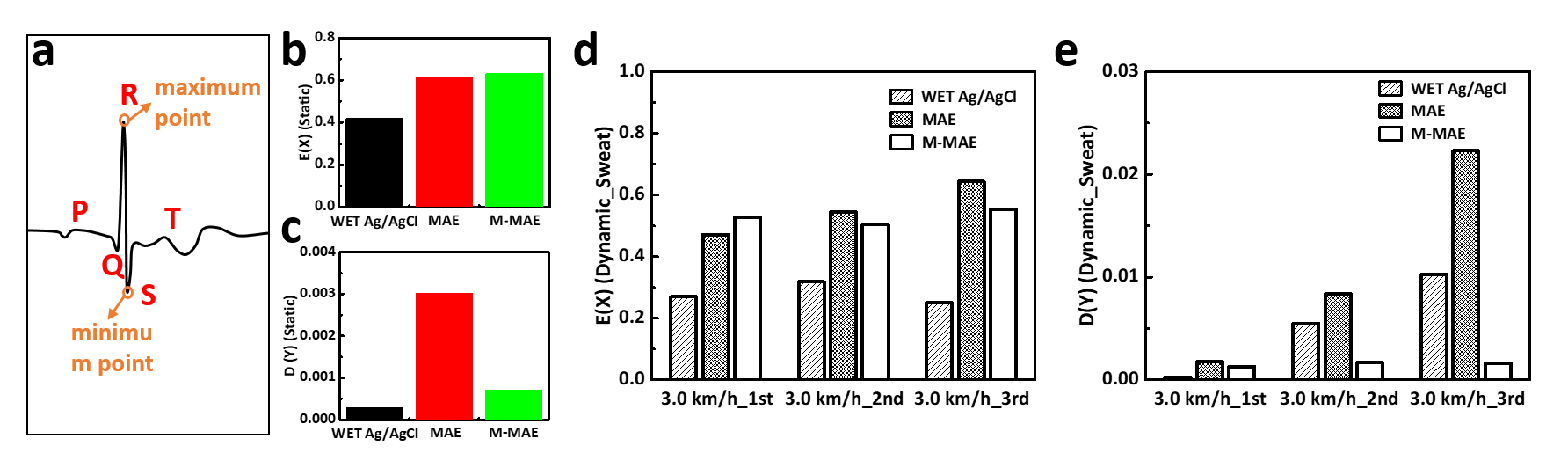
**

**Fig. S5** **a.** One typical wave for ECG. **b.** The expected value for | V_max_-V_min_ | of ECG testing in the static state. **c.** Square deviation for | V_max_-V_min_ | of ECG testing in the static state. **d.** The expected value for | V_max_-V_min_ | of ECG data during the intermittent running test. **e.** Square deviation for | V_max_-V_min_ | of ECG data during the intermittent running test.

The evaluation of performance for these three electrodes on ECG based on the following three criteria:

**1) Expected value for the |V_max_-V_min_|**

The expected value can be calculated from:

$$E\left( X \right)=\frac{1}{N}\sum X$$

where X stands for |V_max_-V_min_| and this value represents the peak-to-peak value in each pulse (fig S5 a). The expected value for ECG data in static state, and intermittent running test with sweat are presented in Fig.S5 b, Fig. S5 d, respectively. The statistical result clearly demonstrates that the electrode with microneedle on top has a larger signal output, which is vital for the doctor to diagnose since irregular pulses can be better presented with a more extensive and clear signal recording.

**2) Square deviation for the** $\frac{\left| \boldsymbol{V}_{\boldsymbol{ma}\boldsymbol{x}}\boldsymbol{+}\boldsymbol{V}_{\boldsymbol{min}} \right|}{\boldsymbol{2}}$

This value can be calculated from the equation below:

$$D\left( Y \right)=\frac{1}{N}\left\{ \sum\left[ Y-E(Y) \right]^{2} \right\}$$

where *Y* stands for $\frac{\left| \boldsymbol{V}_{\boldsymbol{max}}\boldsymbol{+}\boldsymbol{V}_{\boldsymbol{min}} \right|}{\boldsymbol{2}}$ and *E(Y)* stands for the expected value of *Y*. The square deviations are analyzed from the data in fig.6 of the manuscript for both the static and dynamic states to evaluate the whole signal drifting. In the static state tested during a short period, the signal drifting for M-MAE is smaller than the MAE sample, and its performance is also comparable to the traditional wet Ag/AgCl electrode.(**Fig. S5 c**) Furthermore, under the sweaty situation (**Fig S5 e**), with the running intervals increase (labeled as 3.0 km/h_1^st^ to 3.0 km/h_3^rd^), the *D(Y)* of the signal output from the M-MAE sample is much smaller than that of the wet Ag/AgCl electrode and MAE, which also verify the superiority of M-MAE under a sweaty situation during a long period of monitoring.

**3) Has all clear and intact typical waves (QRS, P and T) or not.**


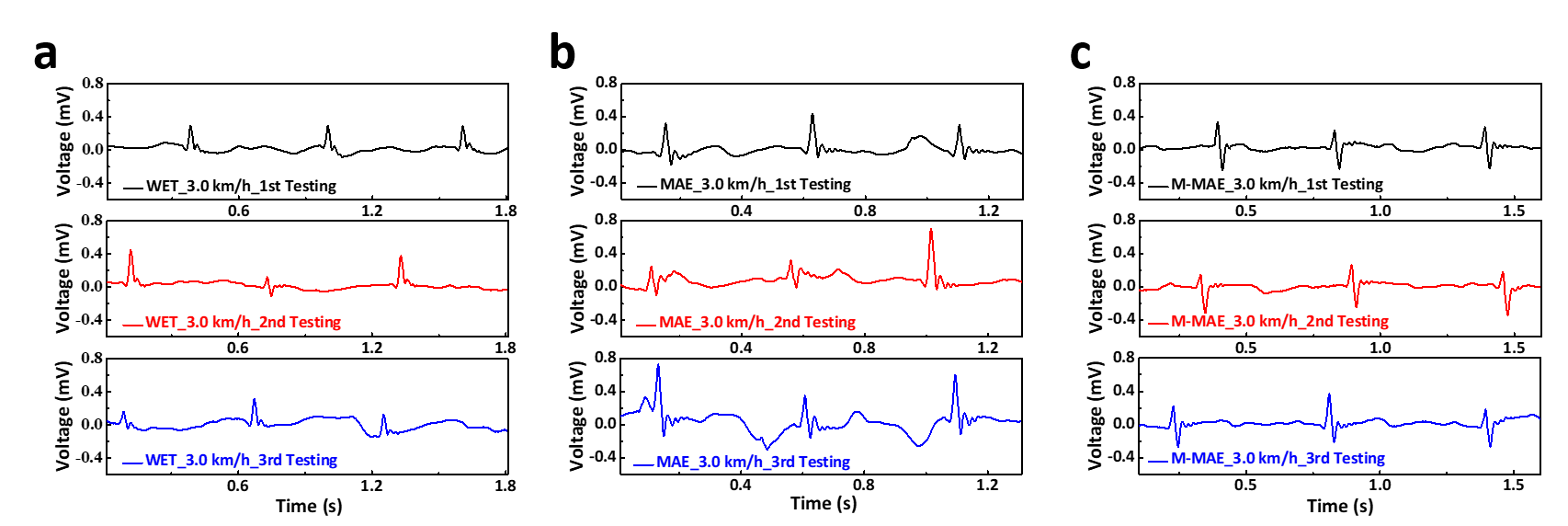


**Fig. S6** Zoomed-in figures for all the first three typical waves ECG data in the dynamic running tests for **a.** wet Ag/AgCl electrode, **b.** MAE and **c.** M-MAE.

The zoomed-in figures of the testing results of Fig.6 are presented here. A much more precise and intact typical wave (especially for the Q and S wave) can be found in the result with M-MAE compared with the other two electrodes from the figures above.

Based on the above three evaluation criteria, the M-MAE sample demonstrates the best ECG testing performance among all three electrodes.


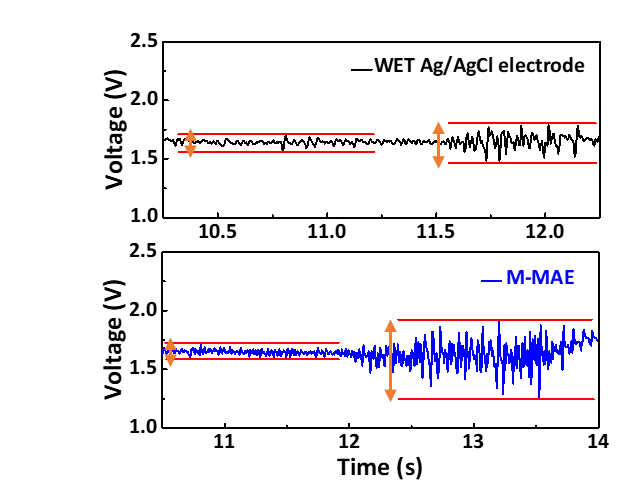


**Fig. S7** Zoomed-in figure for one cycle of EMG testing with wet Ag/AgCl electrode and M-MAE.

Signal to noise ratio (SNR) is calculated based on fig. 6b in the manuscript to compare the level of our desired EMG signal to the background noise. For the signal in volt (V), SNR can be calculated through:

$${SNR}_{dB}=10{log}_{10}\left[ \left( \frac{A_{signal}}{A_{noise}} \right)^{2} \right]=20{log}_{10}\left( \frac{A_{signal}}{A_{noise}} \right)$$

$A_{signal}$ and $A_{noise}$ represent the amplitudes of the desired EMG signal and the noise (fig. S7). Based on the calculation, ${SNR}_{dB\_WET}$ and ${SNR}_{dB\_M\_MAE}$ are 7.79 dB and 15.24 dB, respectively and the noise level for M-MAE is also lower than that of the wet electrode. Better SNR and lower noise level further verify the advantage of M-MAE.

Reference

1. Lv, C., Krishnaraju, D., Konjevod, G., Yu, H. & Jiang, H. Origami based mechanical metamaterials. *Sci. Rep.* **4**, (2014).
